# Therapeutic plasma exchange as rescue therapy in severe sepsis and septic shock: retrospective observational single-centre study of 23 patients

**DOI:** 10.1186/1471-2253-14-24

**Published:** 2014-04-07

**Authors:** Johannes Hadem, Carsten Hafer, Andrea S Schneider, Olaf Wiesner, Gernot Beutel, Thomas Fuehner, Tobias Welte, Marius M Hoeper, Jan T Kielstein

**Affiliations:** 1 Department of Gastroenterology, Hepatology, and Endocrinology, Hannover Medical School, Carl-Neuberg-Strasse 1, D-30625 Hannover, Germany; 2 Department of Nephrology and Hypertension, Hannover Medical School, Carl-Neuberg-Strasse 1, D- 30625 Hannover, Germany; 3 Department of Respiratory Medicine and German Centre of Lung Research (DZL), Hannover Medical School, Carl-Neuberg-Strasse 1, D-30625 Hannover, Germany; 4 Department of Hematology, Hemostasis, Oncology, and Stem-Cell Transplantation, Hannover Medical School, Carl-Neuberg-Strasse 1, D-30625 Hannover, Germany

**Keywords:** Multiorgan dysfunction syndrome, Multiple organ failure, Apheresis, Plasmapheresis, Streptococcal toxic shock syndrome

## Abstract

**Background:**

Several case series and small randomized controlled trials suggest that therapeutic plasma exchange (TPE) improves coagulation, hemodynamics and possibly survival in severe sepsis. However, the exact role of TPE in modern sepsis therapy remains unclear.

**Methods:**

We performed a retrospective observational single-centre study on the use of TPE as rescue therapy in 23 consecutive patients with severe sepsis or septic shock from 2005 to 2012. Main surrogate markers of multiple organ failure (MOF) before, during and after TPE as well as survival rates are reported.

**Results:**

At baseline, mean SOFA score was 13 (standard deviation [SD] 4) and median number of failed organ-systems was 5 (interquartile range [IQR] 4–5). TPEs were performed 3 days (IQR 2–10) after symptom onset and 1 day (IQR 0–8) after ICU admission. The median total exchange volume was 3750 ml (IQR 2500–6000), which corresponded to a mean of 1.5 times (SD 0.9) the individual plasma volume. Fresh frozen plasma was used in all but one treatments as replacement fluid. Net fluid balance decreased significantly within 12 hrs following the first TPE procedure by a median of 720 mL (p = 0.002), irrespective of outcome. Reductions of norepinephrine dose and improvement in cardiac index were observed in individual survivors, but this was not significant for the overall cohort (p = 0.574). Platelet counts decreased irrespective of outcome between days 0 and 2 (p < 0.003), and increased thereafter in many survivors. There was a non-significant trend towards younger age and higher procalcitonin levels among survivors. Nine out of 23 TPE treated patients (39%) survived until ICU discharge (among them 3 patients with baseline SOFA scores of 15, 17, and 20).

**Conclusions:**

Our data suggest that some patients with severe sepsis and septic shock may experience hemodynamic stabilisation by early TPE therapy.

## Background

Sepsis is the most common cause of death in medical intensive care units and the 10th most common cause of death in the United States. The incidence of sepsis has increased over the last two decades with an unchanged mortality rate of approximately 30% for severe sepsis [1] and 40 to 70% for septic shock [2,3]. With withdrawal of activated protein C from the market in 2011, the last approved specific pharmacological intervention for sepsis (aside from antibiotic therapy) was removed from our armentarium, and further treatment modalities are desperately needed. This seems particularly true for the early phase of sepsis where patients are often overwhelmed by a “cytokine storm”.

Sepsis is triggered by exogeneous microbial exposure – so-called pathogen-associated molecular patterns (PAMPs). PAMPs are recognized by pattern-recognition receptors on polymorphonuclear leukocytes and macrophages which initiate microbe-killing systems, production and secretion of cytokines and chemokines, and other proinflammatory mediators [4]. In some cases, the innate immune activation to PAMPs is de-regulated, converting responses that are normally beneficial for fighting infections into excessive damaging inflammation [5]. In addition, certain bacteria such as group A streptococci secrete molecules that down regulate the ability of the host to eliminate bacteria besides other molecules that reduce phagocytic properties or those that activate coagulation [6,7]. The rationale for the use of therapeutic plasma exchange (TPE), a non-selective intervention, is to remove multiple toxic mediators including endotoxins, proinflammatory cytokines and procoagulant factors. Furthermore, depleted plasma factors involved in the homeostasis of microcirculation are replenished by TPE [8]. One such factor could be a disintegrin-like and metalloprotease with thrombospondin type 1 repeats (ADAMTS)-13 that regulates primary hemostasis by proteolyzing von Willebrand factor and is possibly involved in disseminated intravascular coagulation due to severe sepsis [9].

Only very limited data are available regarding TPE for this indication. Two randomized controlled trials with a total of 136 patients examined the impact of TPE on outcome in severe sepsis and septic shock. A promising survival benefit in TPE treated patients in the largest randomized trial (106 patients) with a 28-day mortality rate of 33% in the TPE group compared to 54% in the control group did not remain significant after multiple logistic regression analysis [10]. A smaller trial by Reeves et al. demonstrated a trend towards ameliorated organ failure but no significant difference in mortality [11]. A number of observational studies investigated plasma exchange as rescue therapy in severe sepsis with varying mainly descriptive results [12-24]. Thus, TPE has not yet formed part of international sepsis guidelines [25]. A recent expert consensus statement, however, advocated that patients with refractory septic shock should be considered for extracorporeal blood treatments [26]. Also the 2010 guidelines on the use of therapeutic apheresis in clinical practice [27] list TPE as a 2B (weak recommendation, moderate quality evidence) recommendation in the treatment of sepsis, making the individual clinical and laboratory course of patients along with the available resources the foundation for TPE prescription in sepsis.

In the present manuscript, we report our single-centre experience with TPE as rescue therapy in 23 consecutive patients admitted for severe sepsis or septic shock between 2005 and 2012.

## Methods

We performed this retrospective observational single-centre study in accordance with the ethical guidelines of the 1975 Declaration of Helsinki. The Ethics Committee of the Hannover Medical School waived the need for ethical approval and informed patient consent, as i) data acquisition was retrospective observational within our clinic, ii) data were de-identified, and iii) the study relied on measurements and rescue therapies applied as part of routine care (enquiry #1651-2012).

Severe sepsis was defined by the presence of at least 2 out of 4 SIRS criteria plus suspected infection either by clinical examination, radiological or mirobiological evidence with at least one organ dysfunction. Septic shock was defined as severe sepsis plus systolic arterial pressure < 90 mm Hg or mean arterial pressure < 65 mm Hg over at least 1 h and despite adequate volume resuscitation [25]. None of the patients received hydroxyethylstarch.

All patients were treated based on an in-house protocol adopting the standards recently summarized in the German S-2 k guidelines [28]. Intravenous hydrocortisone was given if adequate fluid resuscitation and vasopressor therapy did not restore hemodynamic stability. Renal replacement therapy was performed as extended hemodialysis using the Genius® batch dialysis system as described elsewhere [29]. TPE as a rescue therapy forms part of our treatment standard in cases with progressive severe sepsis not responding to conventional critical care after weighing its benefits and risks on an individual patient basis. Rescue TPE sessions were stopped either if conventional therapeutic measures alone could effectively ensure clinical stability or if progressive hemodynamic instability, microcirculatory changes, and refractory lactic acidosis led to an end-of-life discussion. We used both, membrane Gambro PF 2000 (polypropylene, 0.4 m^2^; Gambro, Hechingen, Germany) or the Plasmaflow OP by Asahi Medical Co. (polyethylene, 0.5 m^2^; Tokyo, Japan) with Multifiltrate (Fresenius Medical Care, Bad Homburg, Germany) and Octo Nova (Diamed Medizintechnik, Cologne, Germany) and centrifugal (Spectra Optia, Terumo BCT, Denver, CO, USA) technology for the procedure. In every patient we aimed for an exchange volume that equals 1.2 to 1.5 fold of the individual plasma volume. Plasma volume was calculated as follows: Estimated plasma volume (in liters) = 0.07 × weight (kg) × (1-hematocrit) [30]. Removed plasma was substituted with fresh frozen plasma (FFP) in a 1:1 ratio in 22 patients. Only one patient (#10) received 20% albumin diluted 1:4 with an electrolyte solution as replacement fluid. Vascular access was obtained via an indwelling double lumen catheter inserted in the internal jugular vein. In one case TPE was performed over an extracorporeal membrane oxygenation (ECMO) circuit.

Based on patient charts and TPE protocols, organ dysfunction at baseline, as well as during and after the first TPE was assessed [25,31]. Discharge from intensive care unit was the primary outcome. Secondary endpoints were dose of vasopressors as a marker of hemodynamic stability, reductions in net fluid balance as a marker of vascular permeability, and platelet count as a marker of disseminated intravascular coagulation (DIC). We also analyzed need for renal replacement therapy, time from symptom onset to TPE, time from ICU admission to TPE, TPE interval, total number of TPEs performed, as well as exchanged plasma volumes in relation to individual plasma volumes.

Descriptive statistical analysis was carried out with the help of IBM Statistics software, version 21. Kolmogorov-Smirnov test and Shapiro-Wilk test were performed to prove or dismiss suspected normal data distribution. Data were accordingly presented as means (standard deviation, SD), or medians (interquartile range, IQR). Intra-individual changes of net fluid balances, noradrenaline doses, and platelet counts before and after the first TPE were compared by Friedman’s and Wilcoxon test. Presumed parameter variances between survivors and non-survivors were compared by Mann–Whitney-U test, or Student’s T test. Exact Fisher’s test was used to analyse the frequency of occurrence of streptococcal sepsis and immunosuppressive co-morbidities with respect to outcome.

## Results

### Patients’ clinical baseline characteristics and risk factors for acquisition of severe sepsis

We retrospectively identified 23 consecutive patients (14 males, 9 females) with a mean age of 42 years (SD 18) who received TPE for severe sepsis or septic shock. Mean body mass index was 23.5 kg/m^2^ (SD 3.8). Eighteen patients (78%) were in septic shock, and 5 had a severe sepsis. The focus of infection was pneumonia in 14, soft tissue infection in 3, colon perforation with peritonitis in 1, sinusitis in 1, catheter-related in 1, and hepatitis in 1 patient. Two patients had no identifiable infectious focus. Microorganisms and viruses involved (co-infections possible) were streptococci (44%), staphylococci (17%), enterobacteriaceae (13%), Pseudomonas (13%), H1N1-influenza (9%), herpes-virus (9%), and adenovirus (4%). Eighteen patients were at risk of infection due to underlying immunosuppressive therapy, and 5 patients developed severe sepsis without any preceding sign of being immune-compromised (Table 1).

**Table 1:** Septic focus, associated pathogen(s) and risk factors for infection

**Patient**	**Septic focus and associated pathogen(s)**	**Risk factors for infection**
**1**	Septic shock (pneumonia, Staphylococcus aureus)	Microscopic polyangiits, cryoglobulinemia, chronic renal disease
**2**	Severe sepsis (soft tissue infection, Streptococcus pyogenes)	None
**3**	Septic shock	Pancytopenia, cryoglobulinemia
**4**	Septic shock (perianal soft tissue infection, Streptococcus group A)	Excision of an anal tag
**5**	Septic shock (pneumonia, Pseudomonas aeruginosa)	UIP, Sjögren's syndrome, alveolitis, immune complex vasculitis
**6**	Septic shock (parastomal abscess, Peptostreptococcus, Candida) with septic or Infliximab-associated cardiomyopathy	Crohn's disease with anal and parastomal fistulas
**7**	Septic shock (pneumonia, Streptococcus pneumoniae)	Post splenectomy
**8**	Septic shock (pneumonia, Escherichia coli)	None
**9**	Septic shock	Crohn's disease, short bowel syndrome
**10**	Severe sepsis (rhabdomyolysis and pneumonia, Adenovirus, Streptococcus pneumoniae, Staphylococcus aureus)	None
**11**	Severe sepsis (pneumonia, H1N1) with VAHS	Type 2 Diabetes, obesity hypoventilation syndrome
**12**	Septic shock (pneumonia, Staphylococcus haemolyticus, Candida krusei)	MDS with pancytopenia
**13**	Severe sepsis (pneumonia, H1N1, Strepococcus mitis, Serratia marcescens) with VAHS	None
**14**	Severe sepsis (pneumonia)	MDS, secondary AML, PBSCTx
**15**	Septic shock, OPSI (sinusitis, Streptococcus pneumoniae)	Post splenectomy
**16**	Septic shock (pneumonia, Staphylococcus aureus)	Cystic fibrosis, re-double-lung transplantation, diabetes, liver cirrhosis
**17**	Septic shock (pneumonia, Streptococcus pneumoniae)	Multiple myeloma, AL amyloidosis (cardiac, renal), autologous SCTx 4 months ago,
**18**	Septic shock (pneumonia)	Septic granulomatosis
**19**	Septic shock, OPSI (chronic otitis, Streptococcus pneumoniae)	Kidney transplantation, rapidly progressive GN, s/p acute rejection 3 weeks prior with subsequent rituximab treatment
**20**	Septic shock (colon perforation, Pseudomonas aeruginosa, Acinetobacter baumanii, Klebsiella pneumonia)	Crohn’s disease, cachexia
**21**	Septic shock (pneumonia, Streptococcus pneumonia)	COPD
**22**	Septic shock (acute liver failure due to HSV)	Hysterectomy because of uterine myomas
**23**	Septic shock (MOF due to VZV)	Type 2 Diabetes

AML = acute myeloid leukemia, COPD = chronic obstructive pulmonary disease, GN = glomerulonephritis, MDS = myelodysplastic syndrome, MOF = multiple organ failure, OPSI = overwhelming post splenectomy infection, PBSCTx = peripheral blood stem cell transplantation, UIP = usual interstitial pneumonitis, VAHS = virus-associated hemohagocytotic syndrome, VZV = varizella zoster virus.

### Severity of multiple organ failure and outcome

The median number of organs that failed during the ICU stay was 5 (IQR 4–5), mirrored by a mean baseline SOFA score of 13 (SD 4). Only twelve patients (52%) were ventilated on admission, however, 22 patients (96%) needed mechanical ventilation and/or ECMO later during the ICU stay. Median Glasgow coma scale of non-ventilated patients at ICU admission was 14 (IQR 14–15). Norepinephrine was required by 21 (91%) during the ICU stay, vasopressin/terlipressin by 7 (30%), dobutamine by 7 (30%), and levosimendan by 1 patient (4%). Recombinant activated protein C was given to 3 patients (13%), and ECMO (veno-venous or in case of severe cardiomyopathy veno-arterial) was instituted in 8 patients (35%). Baseline pO_2_/F_i_O_2_ was 108 mm Hg (IQR 83–240). Mean baseline creatinine was 221 μmol/l (SD 153). Twenty patients (87%) required renal replacement therapy during their ICU stay. Nine out of 23 patients (39%) survived until ICU discharge (Table 2).

**Table 2:** Inflammation, organ dysfunction and outcome

**Patient**	**Baseline PaO**_**2**_**/F**_**i**_**O**_**2**_**(mmHg)**	**Baseline SOFA score**	**Baseline PCT (μg/l, ULN < 0.5)**	**Ventilatedor on ECMO during ICU stay**	**RRT during ICU stay**	**Organs failed during ICU stay**	**Discharge from ICU**
**1**	140	5	13	Yes	No	4	No
**2**	381	12	54	No	Yes	3	Yes
**3**	89	15	18	Yes	Yes	5	No
**4**	86	17	25	Yes	Yes	5	Yes
**5**	117	11	0,1	Yes	Yes	5	No
**6**	293	11	34	Yes	Yes	6	Yes
**7**	240	16	400	Yes	Yes	5	No
**8**	58	15	94	Yes	Yes	5	Yes
**9**	56	20	12.8	Yes	Yes	5	Yes
**10**	220	4	0.6	Yes	Yes	1	Yes
**11**	107	13	1.8	Yes	Yes	4	No
**12**	102	18	47.8	Yes	Yes	4	No
**13**	200	10	2,5	Yes	No	1	No
**14**	108	7	0.1	Yes	Yes	2	No
**15**	351	9	107	Yes	Yes	5	Yes
**16**	67	11	182	Yes	Yes	4	No
**17**	192	13	30.6	Yes	Yes	5	Yes
**18**	335	11	37	Yes	Yes	4	Yes
**19**	83	16	337	Yes	Yes	5	No
**20**	50	17	3.6	Yes	No	5	No
**21**	80	16	84.7	Yes	Yes	5	No
**22**	283	16	13.7	Yes	Yes	5	No
**23**	93	19	9.6	Yes	Yes	6	No

ECMO = extracorporeal membrane oxygenation, FiO2 = fraction of inspired oxygen, ICU = intensive care unit, paO2 = arterial O2 partial pressure, PCT = procalcitonin, RRT = renal replacement therapy, SOFA = sepsis-related organ failure assessment, ULN = upper limit of normal.

### Dose and adequacy of TPE

A median of 2 TPE therapies (IQR 1–3) were performed and started 3 days (IQR 2–10) after symptom onset, i.e. 1 day (IQR 0–8) after ICU admission. Median total plasma volume exchanged was 3750 mL (IQR 2500–6000), which corresponded to 1.5 (SD 0.9) times the individual plasma volume. All but one TPE therapies used FFP as replacement fluid (median 15 units [IQR 10–24]). Anticoagulation was ensured by heparin in 9 (39%), citrate in 9 (39%) or absent in 5 patients (22%). 21 of 23 patients were treated by membrane technique, 2 by centrifugal technique (Table 3). In those 14 patients who died in the ICU, median time from termination of the first TPE and death was 48 h (IQR 29–162). Two patients (22%) survived to ICU discharge in the heparin anticoagulation group, 5 patients (56%) in the citrate group, and 2 patients (40%) in the no-anticoagulation group.

**Table 3:** Details of plasma exchange therapy

**Patient**	**BMI (kg/m**^**2**^**)**	**Time ICU admission to TPE (days)**	**No. of TPE procedures (day of application)**	**Plasma volume (ml)**	**Total Volume exchange per PV**	**Anti-coagulation**	**Membrane/Centrifuge**
**1**	24.9	0	2 (1,2)	3.70	1.6	Heparin	M
**2**	22.4	1	4 (2,3,4,5)	3.57	4.1	Citrate	M
**3**	17.9	2	1 (3)	2.30	1.1	Citrate	M
**4**	21.6	0	1 (1)	3.17	0.8	Citrate	M
**5**	24.2	8	1 (9)	3.85	0.9	Heparin	M
**6**	15.1	7	3 (9,10,12)	2.07	1.5	Heparin	M
**7**	26.3	0	3 (1,3,6)	4.41	1.8	None	M
**8**	26.0	2	1 (3)	3.20	0.5	None	M
**9**	22.0	2	3 (3,4,5)	2.60	1.9	Citrate	M
**10**	22.0	1	3 (2,3,4)	3.30	3.3	Heparin	M
**11**	29.3	25	2 (25,26)	4.26	0.4	None	M
**12**	30.7	14	3 (14–16)	5.66	1.6	Heparin	M
**13**	22.9	11	2 (11,13)	3.77	1.3	Heparin	M
**14**	18.8	16	1 (16)	3.07	0.8	Heparin	M
**15**	21.4	0	1 (1)	2.60	1.4	None	M
**16**	20.0	2	2 (2,3)	2.15	2.8	Heparin	M
**17**	22.5	1	2 (2,3)	3.05	1.6	Citrate	M
**18**	24.2	0	3 (1,3,4)	3.87	2.6	Citrate	M
**19**	31.2	1	1 (2)	4.38	0.6	None	M
**20**	23.7	0	2 (1,2)	2.83	1.8	Heparin	M
**21**	24.9	1	1 (2)	3.47	0.9	Citrate	C
**22**	24.2	0	1 (1)	3.77	0.8	Citrate	M
**23**	24.2	1	1 (2)	4.20	0.9	Citrate	C

MI = body mass index, FFP = fresh frozen plasma, ICU = intensive care unit, PV = plasma volume,TPE = therapeutic plasmapheresis.

### Influence of TPE on catecholamine dose and net fluid balance

**Figure 1:**
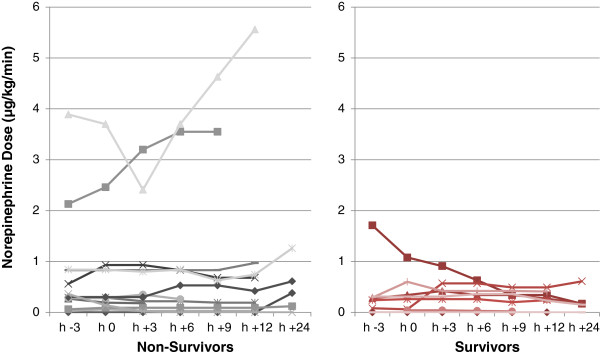
**Norepinephrine dose before, during and after first therapeutic plasma exchange (TPE) in non-survivors and survivors.** Norepinephrine doses as surrogate marker of hemodynamic instability are presented relating to the time of first TPE: 3 hours before initiation of TPE (h-3), initiation of TPE (h0), 3 hours after TPE (h+3), 6 hours after TPE (h+6), 9 hours after TPE (h+9), 12 hours after TPE (h+12), and 24 hours after TPE (h+24).

**Figure 2:**
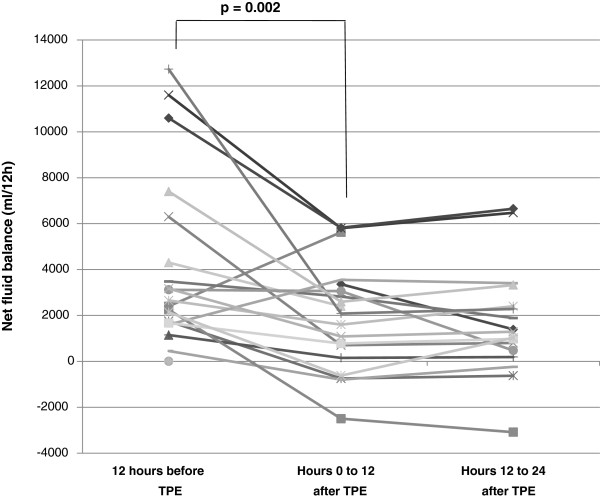
**Net fluid balance before and after first therapeutic plasma exchange (TPE).** Net fluid balances as surrogate marker of septic vascular permeability are presented relating to the time of first TPE: 12 hours preceding TPE, hours 0 to 12 after TPE, and hours 12 to 24 after TPE.

Since mean blood pressures appeared stable during the time periods of plasma exchange, we decided to monitor norepinephrine dose and reduction of net fluid balance compared to the preceding day as surrogate markers of cardiovascular stability. The first TPE procedure neither influenced overall norepinephrine doses (p = 0.574), nor were norepinephrine doses around the first TPE associated with outcome (p > 0.20, Figure 1). However, impressive reductions of norepinephrine doses were seen in individual cases. One example is patient #4 who received her first and only TPE on the day of admission, being associated with a 90% reduction of norepinephrine dose within 24 hours. Another example is patient #6, who developed septic cardiomyopathy with low output syndrome and temporary cardiac arrest on day 7 of her ICU stay but improved substantially after TPE was intiated. In contrast, reductions of net fluid balances by a mean of 720 mL within 12 h following the first TPE were observed in the majority of patients following the initial TPE session (p = 0.002), irrespective of outcome (p > 0.77) (Figure 2).

### Effect of TPE on disseminated intravascular coagulation

**Figure 3:**
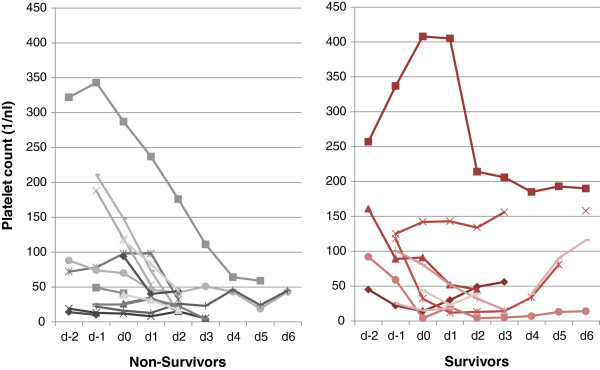
**Platelet count before and after first therapeutic plasma exchange (TPE) in non-survivors and survivors.** Platelet counts as surrogate marker of septic microangiopathy are presented relating to the time of first TPE: 1 day before initiation of TPE (d-1), day of TPE (d0), 1 day after TPE (d+1), 2 days after TPE (d+2), 3 days after TPE (d+3), 4 days after TPE (d+4), 5 days after TPE (d+5), 6 days after TPE (d+6).

Since fibrinogen, prothrombin time and activated partial thromboplastin time are highly influenced by TPE, platelet count was chosen as a surrogate marker of possible influences of TPE on DIC. Time courses of platelet counts between day -2 and day 6 around the first PE therapy were plotted separately for non-survivors and survivors in Figure 3. Platelet counts decreased significantly during the first (p = 0.012) and second (p = 0.023) day following the first TPE procedure, irrespective of outcome (p > 0.31). A stabilisation or increase in platelet count on days 2 to 4 following TPE was observed in 5 out of 9 survivors (56%), but only 1 out of 14 non-survivors (7%).

### Effect of potential predictive parameters on outcome

Survivors and non-survivors did not differ significantly regarding age (p = 0.40), baseline C-reactive protein (p = 0.190), or baseline SOFA score (p = 0.553). The following parameters showed slight differences between survivors and non-survivors, however, without statistical significance: baseline procalcitonin (34 μg/l [IQR 19–74] vs. 13.6 μg/l [IQR 2–109], p = 0.439, upper limit of normal 0.5 μg/l), time from ICU admission to first TPE (1 day [IQR 0–2] vs. 1.5 days [IQR 0–11], p = 0.369), number of TPE sessions (3 [IQR 1–3] vs. 1.5 [IQR 1–2], p = 0.159), and volume exchanged per plasma volume (1600 ml [IQR 1400–2600] vs. 1000 ml [IQR 800–1600], p = 0.159). Four out of 9 patients (44%) who survived and 6 out of 14 patients who died (43%) had been treated with immunosuppressive drugs before the development of severe sepsis. Co-morbidities associated with immunosuppression (e.g. vasculitis, hemophagocytosis, myelodysplastic syndrome, multiple myeloma, recent splenectomy, inflammatory bowel disease, or organ transplantation) were common in survivors (55%) and non-survivors (71%) without significant difference (p = 0.657). Though not significant, septic shock mediated by streptococci (known as streptococcal toxic shock syndrome) tended to be more frequent among survivors than among non-survivors (6 out of 9 [67%] versus 4 out of 14 [29%], p = 0.102).

## Discussion

This retrospective observational study reports the single-centre experience on the use of TPE as rescue therapy in patients with severe sepsis or septic shock and subsequent MOF. Although we often regard RCTs as the summit of evidence-based medicine [32], we should not disregard the role of therapeutic experience in individual patients. The importance of considering other study designs, such as observational studies, in the challenging intensive care unit environment has recently been highlighted [32]. In that sense, our case series focuses on a potential treatment option that has nearly been forgotten during the last 10 years since Busund et al. published the first and largest RCT supporting the use of TPE in septic shock. This trial on 106 patients, 56% of whom had septic shock, seemingly showed a survival benefit in the TPE group which fell short of significance when performing multiple logistic regression [10].

Regrettably, we are still not sure whether TPE exerts any beneficial contribution to the reversal of MOF. Although there are hardly any contraindications of a this rather safe rescue procedure in a near-fatal clinical situation, complications such as urticarial reactions, anaphylactoid reactions, citrate-induced hypocalcemia, catheter-related trauma, clotting, infection, or bleeding as well as transfusion-related lung injury may occur in association with TPE and therefore need to be taken into account [33]. Although TPE implicates significant costs (1267 Euro, i.e. approximately 1577 USD per TPE in Germany), alternative rescue modalities such as adsorption techniques are likely to be even more costly and lack the potential to replenish the organism with removed substances. This consideration and the highly prevalent consumptive coagulopathy led us to use FFP as replacement fluid. Membrane filtration was preferred over centrifugation technique because it was more readily available. However, activation of complement and leukocytes on the artificial membrane are potential disadvantages of the membrane based TPE [34]. Other authors have used similar approaches. Stegmayr et al. performed TPE by continuous centrifugation technique, used liquid stored plasma (1:1) as initial replacement fluid in 90% of sessions, FFP in cases of spontaneous bleeding, and switched to a 5% albumin crystalloid solution after stabilisation or in case of depleted supply pool at the blood bank [24]. Busund et al. used continuous flow plasmapheresis and replaced plasma losses with FFP diluted with 5% albumin in a 1:1 ratio [10]. Reeves et al. used a membrane filtration technique and replaced plasma losses with FFP and a 4% albumin-electrolyte solution mixed in a 1:4 ratio [11].

Compared to other trials in the field of sepsis with MOF, the morbidity in the current case series was very high. The SOAP study on 1177 sepsis patients revealed a mean baseline SOFA score of 6.5 and an ICU-mortality of 27% [35]. The 330 patients with septic shock participating in the trial of Annane et al. displayed a SOFA score of 11 and an ICU-mortality of 45% [36]. Our patients had a median SOFA score of 13 and a median number of 5 failing organs during the ICU stay, resulting in an overall mortality rate of 61%. Although this is comparable to the mortality of patients with 4 or more failing organs in the SOAP study [35], it remains remarkable that three of our most severely ill patients among the survivors of MOF had extraordinarily high baseline SOFA scores of 15,17, and 20, respectively [35,37]. Additionally, patients with advanced MOF often suffer from conditions such as acidosis or bowel ischemia that per se cannot be reversed by TPE therapy. We were, unfortunately, not able to reproduce the very low mortality rates seen by Stegmayr et al. The 76 patients in their retrospective study also suffered from multiple organ failure (88% septic shock, most of them due to streptococci), and the study was comparable to our case series concerning the number of failed organs and TPE procedures performed. However, disease severity was probably lower (66 vs. 87% on renal replacement therapy; 72 vs. 96% on mechanical ventilation or ECMO) [24].

Despite a lack of convincing evidence, it is our impression that several of our patients might not have survived MOF without TPE therapy. This assumption is mainly based on the association of TPE and clinical improvement in these selected cases. As suggested earlier [8] and possibly supported by our data, reversal of thrombocytopenia might be one parameter to monitor TPE-associated improvement. Of note, early post-interventional declines of thrombocyte counts are common [38] and due to loss of platelets in the discarded plasma, filter thrombosis, and hemodilution by infusion of relatively hyperoncotic replacement fluid [33]. Those transient changes should not lead to premature TPE abandonment. Additionally, we have – as others - observed severe septic cardiomyopathy which rapidly and markedly improved after initiation of TPE [21]. Contrary to our expectations and earlier reports [39], vasopressor doses did not improve following TPE in the overall analysis. However, a significantly reduced net fluid balance might - besides an improvement of oncotic pressure by isovolemic protein substitution - indicate that TPE could beneficially effect vascular permeability. In fact, others have described a reversal of fluid shifts from the extravascular compartment into the vessels as an early sign of response even after a single TPE procedure [39]. Timely fluid shifts back into the vascular compartment to achieve a negative fluid balance seem to have major prognostic relevance in critically-ill patients with acute kidney injury, since several studies have shown that fluid overload in these patients is associated with increased mortality [40,41]. Most interestingly, the endothelial angiopoietin-2-Tie ligand-receptor system mediates vascular leakage in sepsis [42], and elevated circulating angiopoietin-2 can be effectively removed by plasma exchange [43].

A comparison of survivors and non-survivors of septic shock might help in the decision when to consider rescue TPE therapy in septic shock. Survivors were felt to be younger, to display higher procalcitonin values, to present at an earlier stage of MOF, to have more plasma volume exchanged, and to receive TPE earlier, although this was not significant due to small patient numbers. The latter observation would be supported by earlier data suggesting that earlier TPE might improve outcome, whereas PE performed > 40 h after presentation is unlikely to be useful in severe septic vasoplegia [14]. Additionally, the high prevalence of streptococcal toxic shock syndrome [44] among survivors might indicate that this entity is particularly amenable to TPE therapy.

We are aware of several important limitations of our study: i) The small patient number and the retrospective observational design are due to the fact that there is only a small window of therapeutic opportunity in fulminant septic shock. Even at a university hospital the minority of such patients are admitted in the very acute phase of overwhelming sepsis where TPE might exert beneficial effects. ii) We presented single-centre data because TPE is far from being established as rescue therapy for septic shock even between tertiary care hospitals, and TPE algorithms and technologies used differ. iii) By analyzing consecutive TPEs instituted for septic shock, we tried to minimize patient selection bias. Still, TPE effects might have been underestimated by including patients with irreversible multiple organ failure. iv) Rescue TPE was instituted on the basis of individual team decisions instead of pre-defined start and stop criteria. Although not significant, those who died received fewer TPEs. It is therefore possible that under-dosing might have lead to underestimation of the efficacy of rescue TPE. v) When analysing the hemodynamic effects of TPE, we focused on the first TPE session, because 10 of our patients (44%) received only one TPE procedure, and we felt that there were too many confounders in this extremely ill patients during the following TPE sessions. This might have underestimated the observed treatment effect.

## Conclusion

In conclusion, our retrospective observational data do neither support nor dismiss the role of TPE in fulminant septic shock on the basis of patient survival or surrogate markers of organ failure. This is probably due to the small patient number and the extremely high morbidity in the current case series that possibly precluded clear TPE effects due to irreversible organ failure. However, TPE might be able to ameliorate DIC and septic cardiomyopathy in selected patients. Most importantly, TPE seems to decrease net fluid balance, possibly by improving vascular permeability.

## Competing interests

The authors declare that they have no competing interests.

## Authors’ contributions

JH designed the study, participated in the care of septic shock patients and drafted the manuscript. CH carried out the plasma exchange therapies and participated in the design of the study. ASS coordinated the therapy of the patients in septic shock and helped to draft the manuscript. OW coordinated the therapy of the patients in septic shock and helped to draft the manuscript. GB coordinated the therapy of the patients in septic shock and helped to draft the manuscript. TF coordinated the therapy of the patients in septic shock and helped to draft the manuscript. TW participated in the design of the study and coordinated the therapy of the patients in septic shock. MMH coordinated the therapy of the patients in septic shock and helped to draft the manuscript. JTK conceived the study and helped to draft the manuscript. All authors read and approved the final manuscript.

## Pre-publication history

The pre-publication history for this paper can be accessed here:

http://www.biomedcentral.com/1471-2253/14/24/prepub
